# Virtual Reality–Based Exposure With 360° Environments for Social Anxiety Disorder: Usability and Feasibility Study

**DOI:** 10.2196/55679

**Published:** 2024-10-21

**Authors:** Mathias Ernst, Stéphane Bouchard, Tonny Andersen, Per Trads Orskov, Kristine Tarp, Mia Beck Lichtenstein

**Affiliations:** 1 Center for Digital Psychiatry Mental Health Services in the Region of Southern Denmark Odense Denmark; 2 Department of Psychology University of Southern Denmark Odense Denmark; 3 Dépt. de Psychoéducation et de Psychologie Université du Québec en Outaouais Gatineau, QC Canada; 4 Centre Intégré de Santé et de Services Sociaux de l'Outaouais Gatineau, QC Canada; 5 The National Research Centre for the Working Environment Copenhagen Denmark

**Keywords:** anxiety, exposure therapy, social anxiety disorder, virtual reality, 360°, mixed methods, interactive

## Abstract

**Background:**

Social anxiety disorder (SAD) is a long-term and overwhelming fear of social situations that can affect work, school, and other daily activities. Although cognitive behavioral therapy is effective, few seek treatment, and many who do start often drop out. This may be due to the component of exposure inherent to cognitive behavioral therapy, where the patient confronts feared stimuli outside the therapist’s office, which they otherwise try to avoid. As an alternative, research has explored the effectiveness of virtual reality (VR)–based exposure therapy with promising results. However, few studies have investigated the feasibility of VR tools using mixed methodologies before assessing their efficacy.

**Objective:**

This study aims to assess the usability, feasibility, and presence of four 360° virtual environments and whether these were able to evoke anxiety in patients with SAD.

**Methods:**

A total of 10 adult participants with SAD and 10 healthy controls were recruited for 1 experimental session (age range 21-32 y; 12/20, 60% male participants). Questionnaire and interview data were collected and analyzed. A mixed methods triangulation design was applied to analyze and compare the data.

**Results:**

Participants with SAD experienced increased anxiety when exposed to VR, and environments were considered relevant and useful as an exposure tool. Participants with SAD reported significantly higher average anxiety levels (*P*=.01) and peak anxiety levels (*P*=.01) compared with controls during exposure; however, significant differences in anxiety when accounting for baseline anxiety levels were only found in 2 of 4 environments (*P*=.01, *P*=.01, *P*=.07, and *P*=.06). While presence scores were acceptable in both groups, participants with SAD scored significantly lower than controls. Qualitative analyses highlight this finding within the SAD group, where some participants experienced presence reduction due to being observed while in VR and in situations with reduced interaction in VR.

**Conclusions:**

VR exposure with 360° videos seems to be useful as a first step of exposure therapy for patients with SAD. Future exploration in the clinical application of VR-based exposure for SAD, as well as means of increasing presence within the virtual environments, may be useful.

## Introduction

### Social Anxiety Disorder

#### Overview

Social anxiety disorder (SAD) is one of the most common mental disorders, with a lifetime prevalence in Western populations of 6.65% to 12.1% [1,2]. SAD is defined as a phobic fear of negative judgment by others [3], including an intense fear of being criticized, ridiculed, humiliated, or ostracized. Physical symptoms of SAD include increased heart rate, excessive sweating, dizziness, and trembling. SAD causes patients to avoid situations where they fear negative judgment, such as shopping, public transportation, social gatherings, or other activities in the public sphere. Individuals with SAD experience a lower quality of life similar to that of outpatients who are depressed [4], and SAD is associated with an array of negative outcomes, such as high unemployment risk, dropout rates, and lower socioeconomic status [5]. Furthermore, individuals with SAD are more likely to develop comorbid disorders, such as depression, other anxiety disorders, or substance abuse disorders [5].

The recommended therapeutic treatment for SAD is cognitive behavioral therapy (CBT) [6,7]. The essential components of CBT include cognitive restructuring and exposure to feared stimuli or situations. Through exposure, patients are confronted with feared social situations they normally avoid [8-10].

However, patients who are aware of their need for treatment may be reluctant to seek it due to feeling ashamed or embarrassed about their symptoms or fear of discussing them with others [11]. Despite readily available and effective treatment, only between one-third and half of people with SAD seek treatment [2,9]. Studies have explored alternative methods of delivering CBT for SAD, aiming to maintain its effectiveness while reducing dropout rates and treatment avoidance [12,13]. One alternative tool in treatment is the use of virtual reality (VR), most commonly used to replace in vivo exposure [14,15].

#### Exposure in VR

VR is defined as the use of computer and behavioral interfaces to simulate the behavior of 3D entities, which interact in real time with each other and with a user immersed through sensorimotor channels. VR is often presented through a head-mounted display (HMD). Content can be either computer-generated images or 360° video recordings. The clinical application of VR in the process of exposure is referred to as VR-based exposure or VR exposure. VR exposure for anxiety disorders has been examined internationally for the past 20 years with overall positive findings [16,17]. Although evidence includes only a few randomized controlled trials [18-21], VR exposure embedded in a CBT framework has proven effective at treating SAD, with meta-analyses showing similar effects as traditional treatment involving in vivo exposure [12,13,15,16] and with an effect that has been found to be persistent [20]. Compared to traditional exposure methods, VR exposure holds several clinical advantages [22,23], such as increased control over situational elements, the opportunity to conduct the treatment in the comfort and security of a therapeutic room, increased motivation, or the opportunity to engage in exposure to situations that are more exaggerated than in vivo. One study has even indicated that individuals with SAD prefer exposure in VR to in vivo exposure due to being too afraid of confronting the real feared objects or situations [24].

An important factor in VR exposure is the sensation of *being there* while in the virtual environment (VE), also known as “presence,” which is instrumental in evoking relevant fear responses needed for VR exposure to be effective [25]. Lee [26] defined presence as a multimodal construct made up of 3 modalities: physical presence, social presence, and self-presence. While the exact relationship between presence and efficacy of VR exposure has yet to be defined, it seems that a certain amount of presence is required to induce a relevant anxiety response in individuals with anxiety disorders and that a certain level of fear is necessary to facilitate exposure [27]. Furthermore, it may be the case that different modalities of presence have different relevance, depending on the targeted disorder. Indeed, social presence may be an overlooked aspect of presence in relation to evoking adequate fear in individuals with SAD.

With the increased use of VR in the treatment of mental disorders, it is increasingly important to include patients in the evaluation of newly developed interventions to determine if the interventions are suitable alternatives to established treatments. Moreover, it is important to understand the details of specific aspects of an intervention to further improve the intervention. This process requires both quantitative measures of usefulness and qualitative measures to gauge factors that may not be covered by any one combination of preexisting questionnaires. Few studies have addressed the clinical considerations of using VR exposure, particularly those that include qualitative feedback from the patients being treated [28,29]. While studies have been made on the efficacy of VR exposure in the treatment of SAD, few studies have applied 360° environments [28,30-32]. Previous studies emphasize the visual and behavioral realism of 360° recordings of real (nondigital avatars) individuals as meaningful in VR exposure for SAD [31,33], as well as cheaper development costs and ease of use [34,35] and similar ability to generate a sense of presence and improve emotional states, when compared to computer-generated environments [34]. However, common criticism of 360° VR includes the lack of immersive properties, such as real-time interaction with the environment, due to the prerecorded nature of the medium, and due to this restriction, it is best applied in a passive or static setting [34,36]. This restriction complicates the development of 360° VR exposure for SAD, as exposure to social situations often requires interaction. Finally, no studies have evaluated the feasibility of these interventions using both qualitative and quantitative measures in a mixed methods design.

In this study, 4 environments were developed and tested on a population of individuals diagnosed with SAD and healthy controls. The environments consisted of multiple scenes with differing complexity and characteristics, using decision tree branching and allowing for real-time adaptation.

### Aim of the Study

The overall aim of this mixed methods study was to evaluate the feasibility of four interactive 360° VR exposure environments for patients with SAD, addressing the following research questions:

1. Are the 360° VEs able to evoke anxiety responses in participants with SAD?
2. Are the VEs relevant to patients with SAD?
3. Are VR exposures perceived as a useful alternative to exposure in vivo?

## Methods

### Design

This study was a feasibility study using semistructured interviews and questionnaire data in a mixed methods design. As the mixed methods model for approaching qualitative and quantitative data, the triangulation design: convergence model was applied [37]. In this model, qualitative and quantitative data on the same phenomenon are collected and analyzed separately. The results are then compared during interpretation in the Discussion section. This interpretation can be designed in different ways. In this study, qualitative and quantitative results are presented with regard to each research question and then compared.

To answer questions pertaining to the VR exposure’s ability to evoke adequate fear responses in participants with SAD, a SAD group and a control group were compared in Subjective Units of Distress Scale (SUDS) ratings administered during the VR exposure. To discuss if the VR exposure is relevant to patients with SAD, estimates of presence were included. Finally, to discuss if the VR exposure was perceived as a viable alternative to exposure in vivo, usability scores were included. To complement the quantitative data, analyses of the qualitative semistructured interview data were conducted to answer each research question. The control group was used in the quantitative section of the study as a point of comparison with the SAD group, while it was not included in the qualitative section of this study, as only qualitative input from the SAD group was assessed as useful to answering the research questions.

A general inductive approach (GIA) [38] was applied to analyze interviews from the SAD group. The GIA seeks to test whether the data are consistent with prior assumptions, theories, or hypotheses identified or constructed by a researcher. The GIA is a systematic procedure for analyzing qualitative data in which the analysis is likely to be guided by specific evaluation objectives with the primary purpose of allowing research findings to emerge from the frequent, dominant, or significant themes inherent in raw data without the restraints imposed by structured methodologies. The GIA was chosen as this study had preestimated objectives from which themes in a semistructured interview guide were developed with the purpose of evaluating the feasibility of the intervention for future trials and implementation. The purpose of the GIA is to condense extensive and diverse raw text data into a concise summary, while establishing clear, transparent, and defensible links between the research objectives and the findings derived from the data. Finally, a model or theory about the underlying structure of experiences or processes that are evident in the text data is developed. The interviews were coded and analyzed using NVivo software (version 14.23.2; Lumivero). To assess trustworthiness, independent parallel coding was used; the first author, ME, and research assistant, Søren Hertz, independently developed categories and themes based on 1 interview. Any inconsistencies were discussed with author KT and merged into a combined set. ME and Søren Hertz then proceeded to code 3 separate interviews, and revisions to the codebook were made in accordance with KT. Finally, ME coded the remaining 4 interviews. The inductive analysis was then performed by ME and KT. The analysis was performed by reading through all the segments coded within each category and theme for each participant. Each paragraph was then further reduced to shorter sentences, describing the core experience within each participant’s relevant experience within each theme. Then, similarities and differences between participants’ experiences were gauged. Then, a list of most to least common experiences was created. Finally, quotes for the most common experiences that best described the collective experience within a theme were highlighted, translated from Danish to English, and used.

### Participants

A total of 21 individuals were invited to participate. The total sample included 20 participants (10 patients with SAD and 10 healthy controls). One individual invited to participate in the SAD group did not show up and was excluded. The age of participants ranged between 21 and 32 years. Of these 20 participants, 8 (40%) identified as female and 12 (60%) identified as male. All participants fulfilled the inclusion criteria of Danish residency and age ≥18 years. Participants in the SAD group fulfilled the diagnostic criteria for SAD (*International Statistical Classification of Diseases, 10th Revision* code F40.1 [3]). Exclusion criteria for all participants were lack of adequate understanding of spoken and written Danish, acute risk of suicide, pervasive developmental disorders, psychosis-related disorders, substance abuse, severe depression, bipolar disorder, severe brain damage, or photosensitive epilepsy. Furthermore, participants in the control group were excluded if they had any anxiety-related disorders, including SAD, agoraphobia, obsessive-compulsive disorder, posttraumatic stress disorder, specific phobias, or generalized anxiety disorder. For the control group, these data were self-reported, and for the SAD group, the referring health professionals verified these data, and self-reports surverys were collected.

### Measures

Measures used in this study, including the Social Interaction Anxiety Scale (SIAS), SUDS, Multimodal Presence Scale (MPS), and System Usability Scale (SUS), are described in the following sections.

#### SIAS Overview

The SIAS [39] is a measure of social phobia symptoms containing 20 items, each of which is answered on a 5-point Likert scale (0-4). The total score range is from 0 to 80. SIAS has also shown good internal reliability, test-retest reliability, and sensitivity to change [39,40] and is moderately correlated with clinician-rated severity of social phobia [41]. SIAS was used before testing.

#### SUDS Overview

Wolpe [42] was first credited with introducing the SUDS, constructed by instructing the patient to “think of the worst anxiety you have ever experienced or can imagine experiencing, and assign to this the number 10 [in this study, 100]. Now think of the state of being absolutely calm and call this zero. Now you have a scale. On this scale how do you rate yourself at this moment*?*” Only the last phrase was used once SUDS had been introduced to participants. Data supports SUDS as a valid global measure of both physical and emotional discomfort [43]. In this study, SUDS was used as a quick global measure of anxiety level during exposure, with the lowest risk of breaking the presence and immersion of the VE.

#### MPS Overview

The MPS [44] is a scale for measuring the psychological construct of “presence” in a VR environment, including 3 modalities of presence: physical presence, self-presence, and social presence; the latter was considered especially important in this study. These modalities were based on the unified understanding of presence as a multidimensional construct, as described by Lee [26]. Two studies were conducted to validate this instrument: the first one included 161 medical students from Denmark [44], and the second one included 118 biology students from Scotland [44].

#### SUS Overview

The SUS [45] is a scale for measuring the usability of developed systems, including technological tools. It has 10 items, which are rated on a 5-point Likert scale (1-5) with a total score of 0 to 100. In an empirical evaluation of the SUS, compiling data from >2300 individual surveys and >200 studies, SUS was found to be a highly robust and versatile tool for usability professionals [46]. In this study, SUS was administered to participants to investigate the usability and acceptability of the developed VR exposure technology. An example of what is measured during SUS is “I think virtual reality was unnecessarily complicated to use.”

#### Semistructured Interview

A semistructured interview was segmented into 2 parts for all participants: one before testing and one after testing. No time limit was established for the interview. The interviews followed an interview guide, which emphasized themes of anxiety, relevance, and usability pertaining to expectations (interview part 1) and experiences (interview part 2). The interview guide contained questions related to each of the 3 themes. Questions were mostly open ended and were not posed if answers to these occurred naturally as a product of open-ended questions (eg, “What are the first thoughts that occur to you after having tried this?”). Interviews were transcribed in NVivo (version 14.23.2) and Word (version 2016; Microsoft Corporation) in full length and anonymized.

While data on heart rate and skin conductance levels were also collected, they were not used in this study.

### Procedure

**Figure 1:**
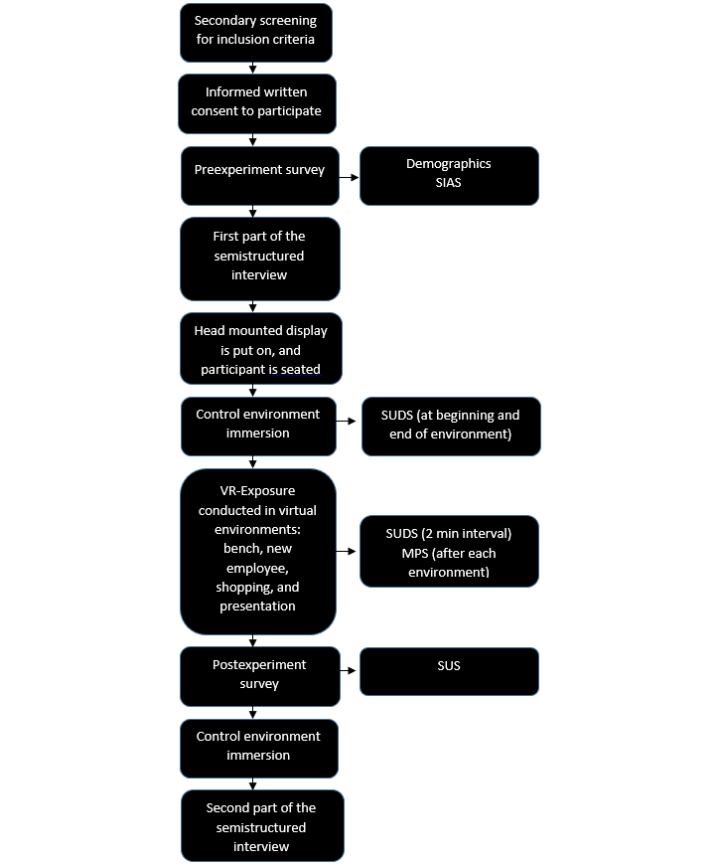
Experiment and dataflow of the feasibility study. MPS: Multimodal Presence Scale; SIAS: Social Interaction Anxiety Scale; SUDS: Subjective Units of Distress Scale; SUS: System Usability Scale; VR: virtual reality.

Recruitment for the SAD group was carried out by referral from health professionals in the Mental Health Services in the Region of Southern Denmark. Controls were recruited using posters at the University of Southern Denmark, local establishments (museums, cafés, and libraries), and social media. Participants were first contacted by phone to gauge their eligibility. For participants who did not wish to have contact over the phone, appointments were made over email, and eligibility was assessed upon arrival. Participants were sent information regarding the purpose of the study before the appointment. Participants were then invited to partake in the experiment, which lasted approximately 2.5 hours. Upon arrival, participants were given a verbal summary of the information they had previously given in writing, and their eligibility was confirmed (Figure 1). Participants were then asked to read and sign the consent form. Afterward, participants filled out a demographic questionnaire, followed by SIAS. Part 1 of the semistructured qualitative interview was initiated. The interview contained predetermined themes of social anxiety, relevance, and usability relating to expectations regarding the VR exposure session, previous experience with both exposure (in vivo) and VR in general. The semistructured qualitative interview was recorded in audio format. Then, a trained research assistant fitted participants with electrodes for recording an electrocardiogram and galvanic skin response. Participants were then seated, and a research assistant fitted them with the HMD. The initial VE was a control environment, depicting a 360° recording of a wintry forest for 5 minutes. Here, participants could become familiar with using the orientation of the headset to move forward, while a psychophysiological baseline was established, and the participant reported SUDS scores when starting the baseline and after 5 minutes. Participants were then presented with 4 exposure environments (described in the following paragraphs). During the exposure environments, SUDS were measured at the start and every 2 minutes throughout the exposure. The environments included idle scenes in which a noninteractive scene looped to allow for either habituation or anxiety increase in a clinical setting. Due to time restraints, all idle scenes were skipped in this experiment, ensuring similar environment duration for all participants and ensuring time for participants to experience all environments. In all environments, choices were made either by the participant or the clinician. After each exposure VE, participants were asked to fill out the MPS. The order in which VEs were presented was reversed for the latter half of participants in both groups. After the 4 exposure VEs, participants were presented with the control VE again. After 5 minutes in the control VE, a research assistant helped participants remove electrodes and HMD. Participants were then asked to fill out the SUS. Then, a final semistructured interview was conducted. Study data were collected and managed using REDCap (Research Electronic Data Capture; Vanderbilt University) [47,48] tools hosted at the Open Patient data Explorative Network, Odense University Hospital, Region of Southern Denmark. REDCap is a secure, web-based software platform designed to support data capture for research studies.

### Materials

The 360° videos were displayed using a VIVE Pro headset (HTC). The headset has a refresh rate of 90 Hz, 1440×1600-pixel resolution per eye, and a field of view of 110°. While the headset permits 6 dfs of tracking, 360° videos only allow for 3 dfs. Videos were recorded using Insta360-InstaOneX (JK Liu) and edited using the game engine Unity 3D (Unity Technologies). iMotions software was used to play and record VEs and visualize real-time physiological data. Physiological data were collected using Biopac products. Scene descriptions were developed by ME and PTO and filmed and edited by collaborative partners from the Maersk McKinney Moeller Institute, University of Southern Denmark.

### VR Environments

To circumvent the linearity of the 360° video format and increase interactivity, environments consisted of 2 types of scenes: “interactive scenes” and “idle” scenes. In interactive scenes, social anxiety–provoking stimulus is presented, and in idle scenes, the environment is simply retained in an idle position. Some idle scenes were also anxiety provoking, such as the crowd during the presentation. Some idle scenes were set to loop after a few minutes, allowing for the possibility of longer or shorter exposure sessions. Furthermore, idle scenes allow patients to stay in the anxiety-provoking situation for as long as needed and allow the clinician control over when, or if, the environment should progress. The participant makes choices by orienting themselves toward a digital overlay and fixing the headset toward the overlay for 2 seconds. The participant is faced with choices regarding movement (marked with an animated arrow overlay) and actions (marked with textboxes, explaining the action). If the participant had no choice when faced with a prompt or question, no digital overlay is presented, and the participant simply answered. Only choices made by the participant were made visible to them. For example, in the shopping environment, a textbox overlay would appear to prompt the participant to either contact an employee or to wait for the employee to leave and grab toilet paper, but when the participant was asked about their previous work experience during the new employee environment, the participant simply answers and the clinician cuts to reactions from the coworkers. Clinicians are faced with choices about whether a scene should progress from an idle scene to an interactive scene or choosing one of the several sequential scenes when >1 branch is available (such as the environment’s reaction to the participant). These choices are made by pressing keys on a keyboard. Environments were developed based on initial expert group interviews and the 4 situational social domains of social phobia identified by Holt et al [49]. Table 1 provides a general overview of the topic, scenes, and purpose of each of the 5 environments (Multimedia Appendix 1 provides full descriptions).

**Table 1:** Overview of the virtual environments used in a virtual reality–based exposure study for social anxiety disorder.

	Bench	New employee	Shopping	Presentation
Topic	Sitting down next to a stranger on a bench	Meeting new coworkers at a staff meeting	Shopping for groceries	Performing a presentation in front of a crowd
Purpose	Exposure to informal interaction with few expectations or social rules Assertiveness in progressive discomfort Direct confrontation	Exposure to informal and formal interaction, switching back and forth Variation in different expressions and body language in “intimate” smaller presentation setting Answering impromptu questions ranging from professional to personal	Exposure to being observed by others Initiating contact Being at the center of attention in a public setting Having an accident in a public setting	Exposure to being observed by others in a formal setting Being at the center of attention Impromptu presentation on any given subject

### Statistics

Quantitative data were analyzed using SPSS (version 28; IBM Corp). Bootstrapped 2-tailed *t* tests (based on 1000 bootstrap samples) with bias-corrected and accelerated CIs on mean scores were run to test for group differences for SIAS, SUDS, MPS, and the social presence modality of the MPS. Finally, graphs illustrating mean SUDS over time (95% CI) were created.

### Ethical Considerations

Upon completion of the intervention, participants would receive a psychological debriefing if needed. The study was carried out in accordance with the Declaration of Helsinki [50]. Participant information was provided in plain language, and written informed consent was obtained from all volunteer participants before data collection. Signed consent forms were sent to all participants who requested a copy through secure web services. Participants were able to withdraw from the project at any time. All data were anonymized before publication. Participants received no compensation. Study data were collected and managed using REDCap [47,48] tools hosted at Open Patient data Explorative Network, Odense University Hospital, Region of Southern Denmark. REDCap is a secure, web-based software platform designed to support data capture for research studies. The study was cleared by the Regional Committees on Health Research Ethics for Southern Denmark (20202000-68). Participation was voluntary, and all participants provided written informed consent.

## Results

### Inductive Thematic Analysis

#### Overview

The interview and the subsequent analysis of the transcribed interviews were structured into 3 categories: anxiety, treatment relevance, and usability. Through means of the GIA, 5 themes were identified in the anxiety category, 4 in the treatment relevance category, and 3 in the usability category (Textbox 1). Categories and themes are presented in the order of sequence in the developed interview guide.

**Categories and themes derived from interviews with participants diagnosed with social anxiety disorder.**

**Anxiety**

- Experiences of anxiety
- Bodily reactions to anxiety
- Anxiety levels
- Anxiety in virtuo versus in vivo
- Self-focused attention

**Treatment relevance**

- Relevance
- Presence
- Interaction
- Realism

**Usability**

- User friendliness
- Technological limitations
- Production quality

#### Anxiety

This category has 5 themes, describing participants’ experiences of anxiety, bodily reactions to anxiety, levels of anxiety, anxiety in virtuo versus in vivo, and self-focused attention. Each theme is presented in detail in the following sections.

#### Experience of Anxiety

All participants had input regarding specific experiences in VR exposure and their associated anxiety responses. The experiences that stood out varied from participant to participant. Participants 1, 2, 7, and 27 all highlighted the fear of not knowing what to say when addressed directly across the different environments, as exemplified by participant 2 in their experience with the new employee environment:

When I had to present myself at the meeting, I had a lot of difficulty saying anything...it just felt overwhelming all of the sudden...the first question they asked me...made me go completely “blank”...it was very uncomfortable.

Furthermore, 2 participants barely spoke aloud, even when prompted, which had a different anxiolytic effect, as experienced by participant 3:

Because I didn’t talk, it just felt like two people were staring me down for no reason...and that is of course a very surreal situation, but it actually gave me a lot, even though it was unreal and almost absurd.

Other participants highlighted the positions in which they were not prompted to engage with the environment and left in waiting positions as being anxiolytic, as explained by participant 27:

I felt the most anxious when...I looked around at all these people I didn’t know, who are all talking to each other, and I was just sitting there, waiting and twiddling my thumbs.

Finally, participants 17, 25, and 27 all had experiences in which eye contact was prominent, as exemplified here by participant 17:

As I stood up there, I could feel, that even though I knew they were really looking at a camera, I felt as if...All eyes were on me.

In summary, the most commonly described anxiety experience occurred in situations when addressed directly. The second most common was the experience of anxiety in situations with eye contact. Finally, experiences of anxiety in waiting positions before speaking aloud and the experience of going “blank” or not being able to talk aloud in the environment were identified as anxiety-provoking aspects for some participants.

#### Bodily Reactions to Anxiety

During the interviews, participants 1, 2, 4, 17, and 25 explained how they had experienced bodily reactions during the VR exposure. These bodily reactions had manifested themselves in what participants normally felt during high arousal in real-world settings, such as a high heart rate, sweaty palms, dry mouth, feeling their hands becoming sweaty, being restless, or experiencing flight response. When asked about how they felt in their body during the exposure, participant 1 explained further:

It’s like getting your heart rate up and such...sweaty palms and such...the things, I normally get, when I get anxiety.

Participant 2 confirmed that they had also experienced the same bodily reactions as they did when they became anxious in the real world:

I am pretty surprised about how much my body reacted...and how difficult it really was...I became especially really dry in the mouth...I could feel my hands and became restless.

In these participant explanations on how their bodies reacted to arousal in VR exposure and what they had noticed about this, we learn that their experiences corresponded to the feelings they had when they felt anxious in the real world. However, we also see that they were surprised about how the level of difficulty in controlling their anxiety, when exposed, could make the body react. In addition, participant 4 described how they tried to address these reactions by relaxing and controlling their breathing. They had also used this technique in other tense situations to avoid drawing attention to themselves.

#### Anxiety Levels

Participants 1, 2, 3, 7, 17, 22, 24, 25, and 27 explained factors that had an impact on the anxiety seeming more or less severe during the VR exposures. The most prominent aspect that reduced anxiety in VR environments was not being contacted, involved, or talked to. Participants explained that they could relax in such a situation but that they would concentrate on not bringing themselves into play, as exemplified here by participant 3:

Just sitting on the bench, she minding her own business and me minding mine, I could relax in that.... And when she was just talking, before it became confronting but when they were just standing around and talking to each other, I would actually think a lot about looking straight ahead and in some way not bring myself into theirs.... So I was thinking a lot about that while I was in it.

In this example, we see that they had experienced less anxiety in a situation where other people were talking and nothing was demanded from them, but also that they tried to divert their attention and ignore what happened around them instead of engaging or seeking confrontation. They used this observer role as a safety behavior, as illustrated by participant 22 when asked about when they felt less aroused:

I think it was the environment about the staff meeting because I felt that I could just sit and observe.... I think that being able to just sit and watch was maybe a kind of protective behavior from my side that I did not need to have any opinion about what they said, and I did not need to make any choices about what should happen next and I could just sit and look at them instead of

interacting

From this example, we learn that simply sharing a physical location with others who were only interacting among themselves made it possible for the patients to disconnect from the situation because there were no social rules or implicit agreement on interaction, and thus, experience reduced anxiety. Another aspect that the patients found to have a protective factor when it came to anxiety arousal was only having superficial contact with other people, as here described by participant 2:

The easiest was to shop.... There was not so much, the most anxiety provoking was when I dropped the eggs.... It was a little bit anxiety provoking but I still felt that it was within the frame of normality of what happens in a shopping center and there were systems and observers to take care of what happened.

Here, we see that they found it a protective factor when there were degrees of normality in the environments and rules about how systems and superficial contact with employees resolved it.

#### Anxiety In Virtuo Versus In Vivo

Participants 1, 2, 3, 4, 7, 17, 22, 24, and 25 had comments comparing their experiences with VR exposure to in vivo exposure. The most common difference was that VR exposure was less anxiety provoking compared to in vivo, while still inducing anxiety. This was experienced by participants 1, 3, 4, 17, 22, and 24 for different reasons. For some, it was simply having the knowledge that the experience was not real (participants 4, 17, and 22), while for others, lack of consequences to their actions played a role, as exemplified here by participant 1:

Some of the things that normally give me anxiety were present here as well, but perhaps not as much as in real life, where I know there are...consequences.

The most experienced difference was the feeling of “simultaneous exposure,” where participants were both exposed to social anxiety in VR while simultaneously exposed to social anxiety in real life from being observed. This further increased anxiety, as exemplified by participant 25:

I kind of knew that I was sitting in this room and...that you were here...if I were actually out shopping in real life, I would have been able to

contact the employee

Here, we see how the task of contacting an employee was perceived as more difficult than in a hypothetical real-life situation due to the knowledge that the experimenter and research assistant were present in the room outside the VE. A similar experience was experienced by participant 3:

When I was prompted to talk out loud...I started thinking a lot about the fact that I was sitting in this room and that people were staring at me...and now they can see that I choose to say nothing...and they probably think something about that.

In summary, the most commonly experienced differences between in vivo exposure and VR exposure were less anxiety comparatively in VR exposure than in vivo due, in some cases, due to the lack of consequences in VR exposure. Another common factor was the knowledge that the environment was not real. Finally, the feeling of “simultaneous exposure” due to both being aware of being observed in the world outside VR exposure and exposed within the VE was another major difference between in vivo exposure and VR exposure.

#### Self-Focused Attention

participants 2, 3, 4, 17, 24, and 25 had experiences pertaining to self-focused attention in VR exposure. All but 1 of these reports increased self-focused attention while immersed. Participants 3 and 4 both reported increased self-focused attention related to the experience of being observed by researchers during the experience, while participant 24 reported quickly forgetting their surroundings outside the immersion. Participant 17 reported that most of his thoughts in the environments revolved around how he was perceived by his surroundings in the immersion:

Most of it [thoughts] were “what do people think about me?”

This was seconded by participant 4 when asked if she noticed anything (reactions) as she was answering questions from the boss in the new employee environment:

No, not really.... Perhaps I did not have everyone’s full attention, I can’t really remember. I mean...Focus was mostly on how I was perceived, and not so much on [how I perceive others]. It is very often like that.

Factors that added to self-focused attention varied. Participant 2 had experienced self-focused attention due to the reduced contact and passive nature of VR exposure when compared to experiences in vivo:

It would be different [in real life] because there is a reduced contact here, so I focus more on myself...being passive...I’m sitting and looking around me, but I can’t move.... I can look them in the eye when they look at me, but...I can’t make any contact other than that. It makes me feel...different from the others in the environment. They can interact. I am sitting here, and I can’t.

Here, participant 2 illustrates how the limitations of VR exposure caused him to feel different from the people he interacted with in the environments. The feeling of being different was tied to limitations on autonomy regarding contact and moving about physically, which in turn caused an increase in self-focused attention.

#### Treatment Relevance

This category has 4 themes, describing participants’ experiences of relevance, presence, interaction, and realism. The 4 themes are presented in further paragraphs.

#### Relevance

Nine participants (2, 3, 4, 7, 17, 22, 24, 25, and 27) provided feedback on the relevance of VR exposure as a tool in the treatment of SAD. All 9 participants found the treatment relevant as a tool in the treatment of SAD. Six of the participants reported that they could see themselves benefit from this VR exposure as part of their own treatment. The most common experience was that the environments were believable as exposure environments they could find themselves in during in vivo exposure. Three participants found the VR exposure relevant as a tool in early-stage intervention for patients who may not be ready for exposure in vivo or who do not have the opportunity to engage with the presented situations VR exposure due to anxiety to approach such situations, as pointed out by participant 17:

I think it would help, but...I think it’s different from person to person, exactly how bad their anxiety is and what exactly their anxiety is in regards to, but I think, in regarding to me, it would be like, well nothing [bad] happened in VR, and that was a representation of what it would be, so I have that experience now. So, I think I would have an easier time taking the chance in the real world and try it out.

Here, the participant identifies how the experience of encountering a feared situation in VR may have contributed to motivation and self-efficacy in pursuing a similar situation in vivo. This is seconded by a different participant 24, who stated the following:

I have always been told in treatment, that having successful experiences with exposure [is important]. So by having a lot of success experiences with increasing difficulty, you get built up step by step until [you think] “okay, now I can do [a], so I can probably also do [b] – and now I can try to go out and do it in real life, because now I have tried it virtually.... So I think at that point, it would help a lot.

Thus, participants saw relevance in the VR exposure, especially in the transferability from the early stages of exposure treatment, to gain confidence in their ability to also attempt in vivo exposure. Finally, 3 participants raised concerns regarding the environments’ “replayability” if they were to be presented with the exact same environment without variation between sessions, as pointed out by participant 25:

I think there would have to be some variation of one kind or another, so it wouldn’t just be like, the same again and again, because then it could really easily become less of a challenge to get through this environment specifically, but it would not have any influence on the anxiety other than in this environment specifically, with VR headset on and so forth.

Here, the participant expresses concern about the repeated use of static environments, questioning their effectiveness in inducing anxiety and their transferability to real-world situations. In summary, participants found VR exposure to be a relevant tool in the treatment of SAD, and most participants could see themselves benefit from VR exposure as part of their own treatment. Relevance in relation to motivation, self-efficacy, and courage to attempt in vivo exposure was experienced, and concerns relating to long-term effects if VR exposure environments remained predictable.

#### Presence

A total of 8 participants reported experiences with the feeling of presence in VR exposure. Five participants (1, 3, 4, 22, and 25) experienced difficulties feeling present in the VEs due to different factors. Three participants describe difficulties letting go of the world outside VR exposure and having their attention switch back and forth between feeling present in VR exposure and the outside world, as described by participant 1:

I was very aware that it was not real, and that you were here in the room as well...sometimes it disappeared, but other times, for example if I were to answer questions [in VR] I was more aware that you were listening as well.

Here, participant 1 raises an important observation regarding the presence of the experimenters in the outside world as a factor that contributed to a decreased sense of presence in the environment. When prompted as to whether anything in the environment prompted the decreased sense of presence, the participant replied the following:

Nah, I just think I knew in advance that it was not real.

Another participant (3) described the experience when approached by a stranger on the bench as follows:

It was not super immersive, but it wasn’t like watching a movie either. It was somewhere between being there and not being there.

Here, the participant emphasized the features of the situation as contributing to only feeling “halfway there.” Finally, 3 participants hypothesized that increased distance to clinicians (using the VR exposure from home) would make it easier to allow themselves to feel present in the environment, and 1 participant experienced feeling more present as time passed during exposure. Finally, 1 participant described curiosity as a limiting factor for experiencing presence and, in some instances, used this curiosity as a distraction to reduce anxiety during exposure.

Three other participants (7, 24, and 27) had experienced that they quickly forgot their surroundings in the world outside VR exposure and felt present in the VEs, as described by participant 24:

I would just sit and talk and have my focus there, so when you weren’t saying anything, I would have a conversation with the VR and completely forgot that you were there...I’d say that I actually completely forgot that I was sitting in this room. I could sometimes get the sense that you were sitting over there by the computer, but I forgot that I was sitting in the middle of the room on this chair.

Here, we see a different example of a participant describing an increased sense of presence in contrast to the previous participants. In summary, some participants experienced difficulties achieving presence in the VEs due to either simultaneous knowledge that the environments were not real, knowing that they were being observed in the world outside VR exposure, or not allowing themselves to feel present as a safety behavior to keep themselves at a distance. Other participants quickly forgot their surroundings in the world outside VR exposure and felt present in the VEs.

#### Autonomy, Influence, Interaction, and Feedback

Nine participants described experiences regarding autonomy, influence, interaction, and feedback within the VE. Most frequently described were different experiences of contrasts to the real-world sensation of either autonomy, influence, interaction, or feedback and how these affected the experience, typically in relation to anxiety levels. Seven participants experienced reduced physical autonomy. These include experiences of not being able to touch anything, not being able to move about freely, and feeling constrained by the situation as a result. This was most commonly experienced in regard to the bench environment, as exemplified by participant 4:

I could not make a choice [to leave] in that situation. I was kind of locked, imprisoned, here on the bench...there was always the question in my mind: “why did I sit here next to her, on the bench? [why was this an action I made?]”

Here, participant 4 describes the experience of reduced autonomy as a decision to engage in the environment feels as if it was made for them, rather than by them—and likewise was the continuous decision to stay put on the bench. Another participant describes similar contrasts with regard to a decreased sense of influence on the situation due to the static nature of the situation and what occurs in it. With regard to interaction, 3 participants experienced that the environments felt the most meaningful, anxiety provoking, and relevant when a sense of real interaction occurred. This sensation was most often achieved when people in VR looked directly at the participant while speaking to them or listening to their responses and when the environment reacted convincingly to their actions. Participant 24 had the following experience:

It is very much up to you what you say...so I thought it was very good that there was [openness in options] and it still fit the situation.

Here, participant 24 explains the importance of the sensation that the environment can hear and respond appropriately to your input. Other participants had conflicting experiences regarding interaction, especially when the interaction was dictated by prompts that were mistimed or did not align with the choices they would make, as experienced by participant 25:

I just stood there, looking at the cooler, and [waiting for a prompt to appear], and until that happened, I would just wait and curiously look around at the surroundings.

Participant 25 then continues to describe how some of the actions taken by prompts in the VR exposure did not feel like him, and thus, were not as anxiety provoking as if he had caused them to happen, such as dropping the eggs in the shopping environment:

The thing with the eggs, I only realized later that: “Oh, that’s what happened.”

In summary, participants experienced limitations to autonomy, influence, interaction, and feedback. Most participants noticed a decrease in autonomy as a product of the limited movement options in a 360° environment. Some participants experienced the sense of believable interaction as the moments in which the environments felt the most relevant to their treatment.

#### Realism

Eight participants had experiences regarding the sense of realism in the VR exposure. Four participants (4, 17, 22, and 24) shared the experience that VR exposure was realistic enough to feel like part of the environment. Four participants expressed surprise at how real it felt (participants 4, 17, 24, and 27), as exemplified by participant 27:

It was surprisingly...Similar...It was like, it really felt like I was there, especially during the presentation. It really felt like I was performing a presentation in front of people I did not know.

Here, participant 27 conveys their experience in comparison with similar experiences of performing presentations in vivo, and they found the 2 experiences to be similar. However, for participant 27, the realness of the environment was especially true for the presentation environment. This was due to fewer cuts between scenes and the ecological validity of the environment. Indeed, these experiences as limiting to the realness of the environments were shared by other participants, as exemplified by 6 participants (participants 3, 4, 7, 22, 24, and 25), who all pointed out the bench environment as being the most unrealistic due to the absurdity of the setting, as explained by participant 3:

And it was of course a very unreal situation, but it actually gave some [anxiety], even though it was unreal and almost absurd...but also, environments that are not realistic may still provide benefits...by turning up.... Making it a little absurd and overgeared...I still think could be very helpful...I thought, if it was real, I would have said: “fine, I’ll go find another bench to sit on” [but here, I could not], so I could definitely feel the awkwardness, it became a bit real.

Here, participant 3 exemplifies how the bench environment was unrealistic due to the situation they were placed in and the manner the storyline progresses. Furthermore, they commented on how this may in fact be beneficial to treatment, as the feeling evoked by the environment is still relevant to the treatment. Finally, 4 participants (4, 24, 17, and 27) pointed out technological limitations affecting their perception of realism in the environments. Three of these participants commented on the “black screen” dividers between scene changes as playing a role in the reduction of environment realism, as experienced by participant 4:

It’s not just that [scene cuts] take me completely out of it [the experience]. It also communicates, the black screen, that something new is about to happen.

Here, participant 4 explained how the dividers may indeed not only affect their presence in the scene but also condition or communicate to the participant that a new “stimulus” is underway, unlike in vivo exposure, where this foresight is not given. In summary, different factors were experienced that led to environments feeling real or unreal. A majority of participants had experienced that environments were either “real enough” or more real than expected. The most common factors that lead to a reduction in realism were “absurd” storylines or technical limitations, most commonly black screen dividers between scene changes.

#### Usability

This category has 3 themes, describing participants’ experiences of user friendliness, technological limitations, and production quality. The 3 themes are presented in detail in further paragraphs.

#### User Friendliness

During the interviews, 10 participants reported experiences with user friendliness throughout the exposure therapy session (participants 1, 2, 3, 4, 7, 17, 22, 24, 25, and 27). One factor that had influenced the participant’s perception of user friendliness was the wear and use of the technical equipment. Participants experienced that it was easy to use and easily understandable. They explained that it was quick and straightforward to have the electrodes from the Biopack put on, as elaborated here by participant 22:

It was fine; I did not find it difficult. I think you could easily do that. Also without other people being present if it was a home assignment. When you have the picture of the model and a short point-by-point instruction on how to put on the electrodes, I think that everybody will easily be able to do it.

Furthermore, they also explained that they did not mind wearing it, as explained here by participant 3:

As soon as the session started, I could easily distract myself from wearing them. I did not think about how it felt to wear them.... In the beginning, I felt the weight of the headset, but then it also quite quickly went away.

Consequently, they had quickly become accustomed to wearing the technology; however, 1 participant reported that it might feel slightly confronting for some people to have it put on by someone else. Moreover, participants felt that it quickly became clear and natural how to orient the direction of the headset to navigate in the environments, but it varied from easy to difficult for them to react to prompts and figure out where and how they were supposed to say something. They emphasized the importance of knowing where the sound buttons are on the headset so that they would be able to adjust the volume without having to ask about it if they were afraid of that.

#### Production Quality Limitations

Seven participants experienced limitations to the production quality of the environments. The most commonly experienced issue, reported by 5 participants (2, 3, 17, 22, and 24), was related to sound. While these were commonly experienced in the baseline environment, others noticed these issues during windy scenes in the bench environment, as pointed out by participant 3:

It was perhaps just the first [environment] where there was a lot of wind. The microphone should have probably had a proper muffler.

Here, participant 3 exemplifies audio limitations as part of the production quality that could have improved the realism of the environment. The second most popular, experienced by 3 participants (2, 3, and 4), was the dialogue and interaction in the scenes, which were sometimes unconvincing or not believable. This is explained by participant 4:

I mean, of course, it’s acting, isn’t it?...The way they say things and stuff [seems rehearsed] and not quite genuine reactions they have. Not that it feels forced...It’s something you can overlook. It’s not that it ruins the experience.

Here, participant 4 identifies a possibility for improvement in the convincingness of the acting as an element that could improve the overall experience of the VR exposure. Finally, other less frequently experienced limitations to production quality included digital overlays as being distracting, distracting sounds from children (during the new employee environment), and reuse of the same actors across multiple environments. In summary, the most common experiences relating to production quality limitations were regarding poor audio quality and, at times, the degree to which acting in the scenes was considered convincing.

#### Technological Limitations

Seven participants experienced technical setup elements that reminded them that they were in a VR environment, pulling them out of the immersive experience (participants 2, 3, 4, 17, 24, 25, and 27). Here, examples that made the experience more unnatural were that they had to orient the direction of the headset to move on in the environments, they could see the transition between loops in the videos, they could feel the weight of and wires from the headset, they could not hear their own voice when speaking, and they could feel the galvanic skin response electrodes on their fingers. The most common technological limitation shared by 4 participants (participant 2, 3, 4, and 27) were the cuts and jumps in transitions between scenes. This included both the dark screen dividers between scenes and teleportation in movement, as experienced by participant 2:

It would be nice if there weren’t any cuts or jumps at all.

Second most common, experienced by 2 participants (3 and 27), were resolution rates, at times becoming pixilated, affecting the presence or ability to properly see faces at a distance. This was exemplified by participant 3:

The resolution could have been higher. I know there are limitations to the headset as well, but it seemed a bit pixilated at times. Especially in environment 2 [New Employee], there were moments where I could have had eye contact [with coworkers], but their faces were too blurry.

Here, participant 3 identifies resolution quality as a technological limitation, which may affect relevant aspects of exposure for SAD, namely, eye contact. Finally, other less common technological limitations included distortions in image quality when looking up or down or where the image was fused within the recording sphere (participant 2) and a narrow field of view (participant 3). In summary, several technological limitations were identified by participants, most frequently involving the scene transitions and movement options, as well as variations in resolution quality.

### Quantitative Results

#### Overview

**Figure 2:**
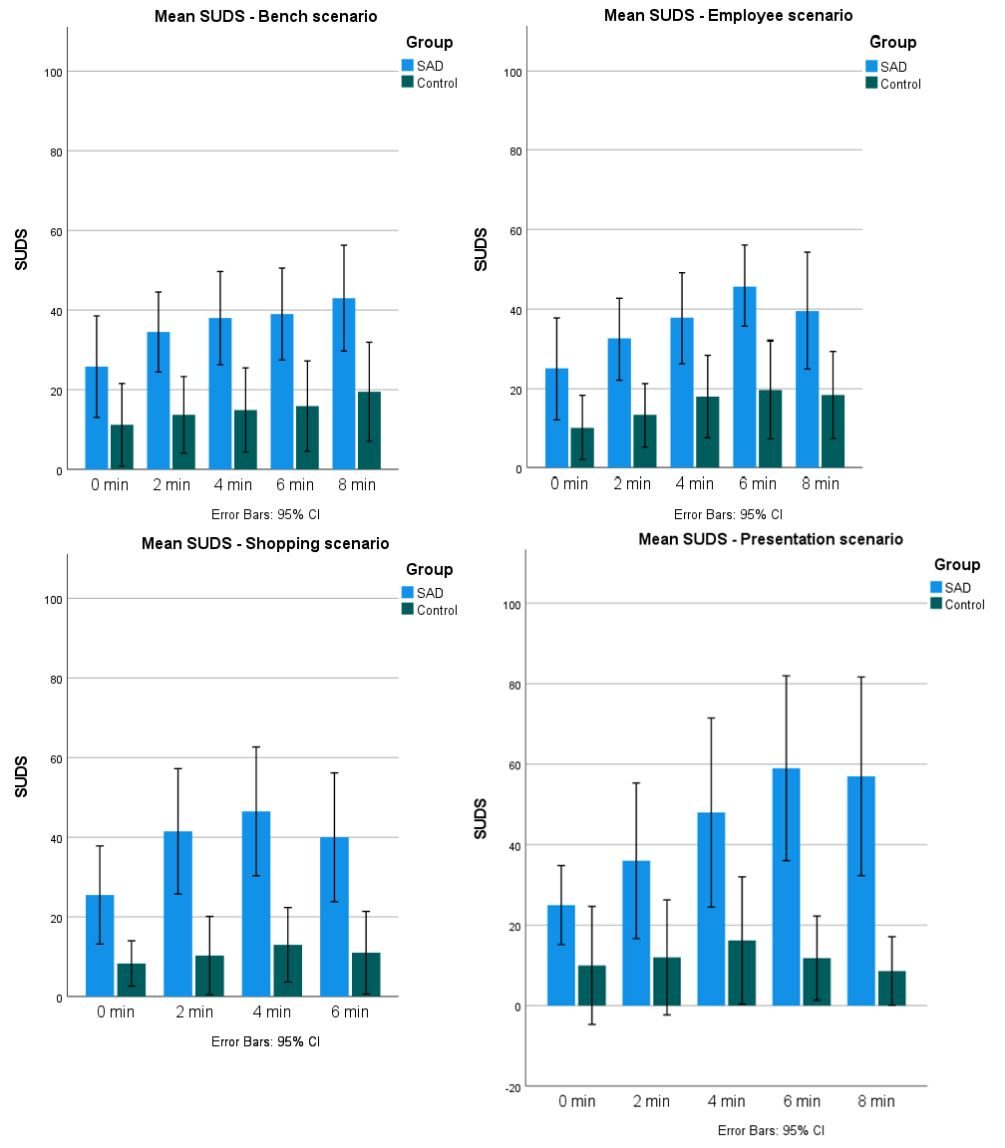
A visual comparison of anxiety ratings over time in each of the 4 environments, based on clinical trials comparing the social anxiety disorder (SAD) group (n=10) and control group (n=10) at the Center for Digital Psychiatry, Odense, Denmark, from April 2021 to July 2022. The shopping environment only included measurements until 6 minutes due to missing data. SUDS: Subjective Units of Distress Scale.

Initially, independent *t* tests were performed on relevant data pertaining to total scores of anxiety and presence across all environments. To account for the low sample size, 2-tailed *t* tests were bootstrapped (based on 1000 bootstrap samples), and CIs were bias corrected and family-wise corrected based on 2 factors: anxiety and presence (Table 2). Anxiety contains 3 categories (SUDS total, SUDS total peak, and SUDS total peak-baseline); thus, the significance level was set to 0.0167 (0.05/3=0.0167). Presence contains 2 categories (MPS total and MPS total [social presence]); thus, a significance level was set to 0.025 (0.05/2=0.025). SIAS is considered a pretrial descriptive measure and, thus, is not included in either factor. Then, factors with significant differences were further explored in environment-specific detail (Table 3). Finally, means for each group at each time point in each environment are presented (Figure 2).

**Table 2:** Family-wise corrected independent bootstrapped t test with bias-corrected CIs, based on clinical trials comparing the social anxiety disorder (SAD) group (n=10) and control group (n=10) at the Center for Digital Psychiatry, Odense, Denmark, from April 2021 to July 2022.

	SAD			Control			*P* value (bootstrapped, equal variances not assumed)
	Scores, mean (SD)	BCa^a^ 95% CI mean (bootstrapped)	Scores, mean (SD)		BCa 95% CI mean (bootstrapped)		
SIAS^b^	55.40 (10.31)	48.53-61.33	12.1 (13.22)		4.67-20.60	<.001^c^	
SUDS^d^ total	39.15 (15.85)	28.71-47.64	14.80 (12.72)		7.45-23.25	.01^c^	
SUDS total peak	50.20 (17.24)	37.32-59.58	20.08 (14.86)		10.82-29.61	.01^c^	
SUDS total peak-baseline	29.95 (13.55)	20.47-39.35	13.48 (10.82)		6.35-20.50	.01^c^	
MPS^e^ total	47.13 (8.66)	41.22-53.46	58.83 (7.72)		54.17-62.95	.01^c^	
MPS total (social presence)	3.47 (0.63)	3.01-3.89	4.10 (0.46)		3.82-4.35	.04	

^a^BCa: bias corrected and accelerated.

^b^SIAS: Social Interaction Anxiety Scale.

^c^Significant difference.

^d^SUDS: Subjective Units of Distress Scale.

^e^MPS: Multimodal Presence Scale.

**Table 3:** Environment-specific exploration based on significant differences in total scores between the social anxiety disorder (SAD) group (n=10) and the control group (n=10). This comparison is based on clinical trials conducted at the Center for Digital Psychiatry, Odense, Denmark, from April 2021 to July 2022.

	SAD			Control			*P* value (bootstrapped, equal variances not assumed)
	Scores, mean (SD)	BCa^a^ 95% CI mean (bootstrapped)	Scores, mean (SD)		BCa 95% CI mean (bootstrapped)		
SUDS^b^ bench	36.36 (15.45)	26.38-45.51	15.14 (14.96)		6.67-24.66	.01^c^	
SUDS new employee	36.12 (15.38)	27.27-44.86	15.88 (13.37)		8.01-24.15	.01^c^	
SUDS shopping	38.55 (19.86)	26.58-49.08	10.94 (12.36)		4.18-18.84	.01^c^	
SUDS presentation	45.57 (20.02)	31.31-56.36	17.25 (11.45)		9.87-24.56	.01^c^	
SUDS bench peak	46 (15.95)	35.39-55	20.3 (16.89)		10.35-31.44	.01^c^	
SUDS new employee peak	47.3 (15.83)	37.61-56.75	21.6 (16.73)		11.20-32.23	.01^c^	
SUDS shopping peak	49 (23.43)	35-61.61	15 (14.64)		6.89-23.73	.01^c^	
SUDS presentation peak	58.5 (25.39)	38.06-72.80	23.4 (13.25)		14.71-31.66	.01^c^	
SUDS bench peak–baseline	25.75 (12.75)	18.57-34.01	13.7 (12.95)		5.31-22.08	.07	
SUDS new employee peak-baseline	27.05 (12.21)	19.55-35.25	15 (13.35)		6.14-23.57	.06	
SUDS shopping peak-baseline	28.75 (20.01)	17.03-41.88	8.4 (10.46)		2.32-14.89	.02^c^	
SUDS presentation peak-baseline	38.25 (23.06)	21.52-54.14	16.8 (9.24)		9.99-22.87	.03^c^	
MPS^d^ bench	44.8 (10.41)	38.17-51.77	58.8 (8.47)		54.59-63.16	.01^c^	
MPS new employee	49.1 (9.64)	42.41-56.11	59 (8.84)		53.83-63.65	.03^c^	
MPS shopping	44.4 (10.52)	37.41-51.44	57.6 (9.59)		52.60-62.56	.02^c^	
MPS presentation	50.2 (9.45)	44-56.56	59.9 (11.04)		52.23-66.78	.05	

^a^BCa: bias corrected and accelerated.

^b^SUDS: Subjective Units of Distress Scale.

^c^Significant difference.

^d^MPS: Multimodal Presence Scale.

#### SIAS Scores

With cutoff scores of 34 to indicate SAD, all participants in the SAD group scored above the cutoff for SAD (mean 55.40, SD 10.31; range 48.53-61.33). For controls, all but 1 participant scored below the cutoff for SAD (mean 12.1; range 4.67-20.60). There was a significant difference in SIAS score between the 2 groups (*P*<.001).

#### SUDS Scores

Scores ranged from 0 to 85 in the SAD group and 0 to 50 in the control group. The results presented include all SUDS scores from all time points for each environment (Figure 2 shows a visual comparison of each time point). Significant differences between groups were found for all 3 categories (SUDS total, SUDS total peak, and SUDS total peak–baseline; Table 2). In the environment-specific exploration of SUDS, each of the categories revealed significant differences, except for measurements regarding baseline minus total peak (SUDS total peak–baseline) in the cases of the bench environment (SAD: mean 25.75; range 18.57-34.01 and control: mean 13.70; range 5.31-22.08; *P*=.08) and the new employee environment (SAD: mean 27.05; range 19.55-35.25 and control: mean 15; range 6.14-23.57; *P*=.06; Table 3).

#### MPS Scores

Significant differences were found between the 2 groups in MPS total scores; however, no significant difference was found between groups when isolating social presence (SAD: mean 3.47; range 3.01-3.89 and control: mean 4.10; range 3.82-4.35; *P*=.04), and thus, this measure was not included in subsequent exploratory findings (for further details on this finding, contact ME).

In the environment-specific exploration of MPS (Table 3), a significant difference between groups was found in 3 of the 4 environments (bench, new employee, and shopping), with consistently higher mean scores in the control group. No significant difference was found between groups in the presentation environment (SAD: mean 50.20; range 44-56.56 and control: mean 59.90; range 52.23-66.78; *P*=.05).

#### SUS Scores

SUS is scored on a scale from 0 to 100. The scores are then translated into 1 of 7 adjective ratings: worst imaginable, awful, poor, okay, good, excellent, and best imaginable. Scores ranged from 65 to 90 in the SAD group, with an average score of 78.75. The control group ranged from 72.5 to 97.5, with an average score of 87. The adjective ratings of the average scores for the SAD group translate as *good* and for the control group as *excellent*.

## Discussion

### Overview

The first aim of the study was to investigate if the VR environments were able to evoke adequate subjective fear responses in participants with SAD. Both analyses supported the fear-evoking capabilities of VR exposure. To investigate this aim, the thematic category of *anxiety* was established. Using the GIA, 5 themes emerged: *experiences of anxiety*, *bodily reactions to anxiety*, *anxiety levels*, *anxiety in virtuo versus in vivo*, and *self-focused attention*. During the interviews, participants with SAD reported experiencing fear responses in all the environments. This is also apparent in the quantitative data, where participants with SAD reported significantly higher average SUDS scores and peak anxiety when compared to the control group. The qualitative analysis found that participants often perceived their anxiety levels as lower in VR compared to what they would have experienced in real life. While participants merely reflected on this difference, as no in vivo setting was presented during this study, this difference has also been found in other studies [51]. Participants explain that one of the reasons for this was the preemptive knowledge that the situations were not real, reducing the potential lasting consequences of actions. While the exact relationship between fear and presence is still discussed, increases in emotional engagement (eg, fear) are usually accompanied by increases in presence [25]. The most common factors for situations in which fear occurred were during moments in which they were prompted to interact with the environment, either by making contact, having eye contact, speaking out loud, or being at the center of attention, such as in the presentation, which showed the highest mean anxiety score of all the environments (SUDS score=46.67) and mean peak anxiety (SUDS score=58.50). Fear decreased in moments where participants could assume an idle position and detach from the experience, such as the bench environment, which showed the lowest average SUDS score (SUDS score=35.91) and the lowest mean peak anxiety (SUDS score=46).

Bodily reactions, such as increased heart rate and sweatiness, were reported as well, testifying to the ability of the exposure environments to evoke adequate fear. Finally, participants report experiences of anxiety not only in relation to the environments but also to the simultaneous exposure of the experimental setting itself, which is also apparent in the difference in SUDS scores between the SAD group (SUDS score=18.75) and the control group (SUDS score=6.08) during the baseline recording. When accounting for this difference, significant differences in SUDS, was found in 2 (shopping and presentation) of the 4 environments. This may be due to the differing nature of the environments, with the presentation and shopping environments being inherently task based and the bench and new employee environments allowing for idle time in between interaction. While no inferences could be drawn regarding the difference over time between groups, the most anxiety-provoking VE for the participants with SAD was the presentation. In this VE, participants were prompted to make a presentation on an unprepared subject in front of a crowd of people. Most participants selected the option of standing up in front of the crowd. It makes sense that this VE was the most anxiety provoking, as the performance task is considered the most anxiety inducing for individuals with SAD. Combining the results from the qualitative analysis and the data from the quantitative analysis, we can see that the environments were indeed able to evoke anxiety in participants with SAD. In addition, exposure to environments that actively engage participants and do not allow for idling is the most anxiety provoking. In this study, participants did not receive external tasks from a therapist, but they solely relied on the VEs’ exposure capabilities. In clinical use, the bench environment and new employee environment may show increased anxiety-provoking capabilities if used properly by a therapist to prompt engagement and introduce tasks.

The second aim was to investigate if VEs were perceived as relevant to the treatment of SAD. To investigate this further, the thematic category of *treatment relevance* was established. Using the GIA, 4 themes emerged: *relevance*, *presence*, *interaction*, and *realism*. There was a consensus among the participants with SAD that the environments were relevant to situations they would have difficulties being in in vivo. In the quantitative analysis, we saw a significant difference between the level of anxiety felt by the SAD group and the level of anxiety felt by the control group at all time points across all exposure environments, with the SAD group consistently scoring higher on SUDS. This might indicate that the exposure environments were relevant because they were able elicit higher levels of SUDS in the patients with SAD compared to controls. Participants reported either being able to see themselves benefit from VR exposure or see VR exposure applied to other individuals with SAD as an early exposure exercise before in vivo exposure. There were mixed experiences of feeling present in the environments, as some participants experienced difficulties “letting go” of the simultaneous knowledge that they were being observed by experimenters. Furthermore, some factors within the environments were identified as either increasing or decreasing presence. Participants felt the most present when interacting meaningfully and realistically with the environment, such as during the presentation or when answering questions, and they felt the least present when they could passively sit and observe the environment. In the development of VE for exposure, these findings point to the importance of accurately mimicking bidirectional social interaction and social presence, as pointed out by Felnhofer et al [52]. Other situations in which autonomy was lower, such as not being able to move, behave, or interact with the environment in the preferred manner, also reduced the sense of presence. Interaction was deemed meaningful when appropriate timing and responses occurred in the environment. Finally, environments were generally perceived as realistic. Factors such as sound issues and resolution in some situations reduced the realism of the VR exposure. Moreover, the bench environment was estimated as the least realistic situation due to the “absurd” nature of the situation and interaction. To optimize presence, it is important to carefully construct the storyline and interactions within the environment.

The quantitative data used to investigate this aim consisted of MPS surveys measured for each of the environments. Mean presence scores were higher for the control group across the environments, and this difference was significant for all environments, except for the presentation. The comparatively lower presence scores in the SAD group synergize with frequent experiences reported in the qualitative analysis regarding difficulties among the SAD group in “letting go” of the simultaneous knowledge of being observed by experimenters. Thus, differences in presence may not be explained by the VEs themselves but rather by their combination with the confounding variable of being observed during the experiment and may increase as a therapeutic alliance is built with the practitioner.

The third aim was to investigate if the VR exposure was perceived as a useful alternative to exposure in vivo among patients with SAD. Both analyses found support for this. To investigate this aim, the category of *usability* was established. Using the GIA, 3 themes were identified: *user friendliness*, *technological limitations*, and *production quality*. Participants experienced very few difficulties navigating the environments and found the tool intuitive to use. Some technological limitations were identified, such as being aware of sensors and wires, a narrow field of view, and audio quality. Some participants had comments regarding situations in which the production quality could be improved, such as situations in which rehearsed lines were obvious or where digital overlay affected their sense of presence and realism in the environments.

The quantitative data used to investigate this aim consisted of SUS surveys. Overall, SUS scores were rated *good* for the SAD group and *excellent* for the control group across all environments.

The use of 360° environments has several drawbacks regarding certain aspects of presence (especially self-presence) and autonomy limitations in movement and behavior due to 3 dfs compared to 6 dfs in animated environments. However, for this study, 360° environments were chosen due to ease of use and reduced developmental costs, the latter allowing a wider array of social environments to be developed. Furthermore, while computer-generated VR is advancing rapidly, there is currently no consensus on whether it or 360° VR offers superior realism and presence. However, one study found no difference in presence [34], and other 360° VR exposure studies for SAD emphasize realism as a deciding factor in choosing 360° VR [31,33]. Future studies should look into the ecological validity of animated versus 360° recorded environments and the transferability of VR exposure experiences to real-world interaction in patients with SAD or similar disorders in which the object of fear relies on social interaction.

### Strengths and Limitations

This study has several strengths. As mentioned earlier, a clear challenge in using 360° VR in VR exposure for SAD is the lack of interaction due to the prerecorded nature of the medium. Indeed, several studies use 360° VR exposure for public speaking scenarios, where interaction with the audience is limited and thus easier to simulate. However, there are fewer studies that attempt to simulate more dynamic back-and-forth communication. This study is novel in the sense that it attempts to mitigate this weakness by introducing immersive interaction in 360° environments, allowing a wider array of social situations to be experienced in 360° VR, along with increased replayability. This has potential clinical upsides, as it allows for less rigid exposure settings and intensity and, thus, higher flexibility in treatment. Furthermore, while a single VR exposure environment may be applicable to reduce SAD symptoms, an array of differing and flexible social situations may provide higher ecological validity to the patient (eg, exposure to less structured social situations).

Second, the application of a mixed methods approach allowed us to gauge the feasibility of the virtual exposure environments from different angles, allowing for input and findings that no single method could have provided. Second, the study included participants already diagnosed with SAD from an outpatient psychiatric clinic rather than relying on self-reported SAD symptoms alone, and an active control group was included to compare against. Third, the qualitative process has the strength of including several researchers in the coding process. Finally, the study included an investigation of social presence as a relevant subscale of exposure to SAD.

The study also has limitations. First, the qualitative analyses could have been strengthened by including stakeholder checks and participants in the qualitative analysis process. Another limitation of the study design is that we are unable to comment on the effects of the treatment when compared to treatment as usual. For this, the study would need to include a larger sample and have several treatment sessions. This study has been started, the protocol has been published, and the study is currently ongoing [53].

Furthermore, the design choice of this study only allowed for participants to experience the environments in a single session, with the purpose of evaluating the feasibility of the developed VR exposure. Thus, this study does not provide any longitudinal effect estimates of VR exposure as a tool in treatment. Due to the standardized application of the VEs, participants did not experience the flexible, creative means of application one may include as part of the VR exposure treatment (eg, impromptu tasks tailored to each patient or prolonged exposure during idle scenes in awkward or uncomfortable situations).

SUDS measurements were assessed every 2 minutes with an allowed error margin of 10 seconds to assure participants were not interrupted during prompts in the environments. Moreover, while idle scenes without interaction were skipped, participants spent a differing amount of time replying to prompts or performing tasks, causing measurements to be made at different time points in the environments. Thus, measurements were not sensitive to potential environmental triggers.

### Conclusions

This study investigated the feasibility of a 360° VR exposure tool for SAD, which included 4 different VEs: “bench,” “new employee,” “shopping,” and “presentation.” Overall, the VR exposure tool was feasible for use in exposure therapy for patients with SAD. The tool was deemed the most relevant when participants were engaged completely with the environment. Such engagement can occur either naturally within the environment or by establishing tasks or goals preemptively to exposure. To fully use the VR exposure in a clinical setting, it is important that the tool be used actively by the clinician and patient rather than passively as an immersive movie.

The effectiveness of the tool must be tested with a larger sample size in a randomized controlled setting. This study is currently ongoing.

## Appendix

Multimedia Appendix 1 Environment descriptions and figures.

## Abbreviations

CBT: cognitive behavioral therapy
GIA: general inductive approach
HMD: head-mounted display
MPS: Multimodal Presence Scale
REDCap: Research Electronic Data Capture
SAD: social anxiety disorder
SIAS: Social Interaction Anxiety Scale
SUDS: Subjective Units of Distress Scale
SUS: System Usability Scale
VE: virtual environment
VR: virtual reality
