# Supplementary material for: Virtual Reality–Based Exposure With 360° Environments for Social Anxiety Disorder: Usability and Feasibility Study
Source: JMIR Form Res. 2024 Oct 21;8:e55679. doi: 10.2196/55679 (PMC11535800; doi:10.2196/55679)
Supplement: Multimedia Appendix 1 [file formative_v8i1e55679_app1.docx]

# Appendix

## Environment descriptions

*Baseline scenario (5 mins) before and after exposure:*In this scenario, the participant is immersed in a wintery forest with no other people around. The participant can look around and move forward, through the forest, by orienting the HMD towards an animated overlay depicting an arrow for 2 seconds (figure 1).

*Bench:*
In this VE, the participant is in a park, standing across from a bench. A stranger is seated on the bench. After a few seconds, an animated arrow overlay appears, pointing towards the bench (figure 2). If fixated on for 2 seconds, the scene cuts to a seated position on the bench next to the stranger. Once the participant sits down, the stranger briefly says ‘hello’ and then returns to looking at her phone. The scenario then cuts to a looping idle scene during which the stranger keeps looking at her phone. Idle scenes were not reused, but recorded in succession of each interaction scene. The clinician decides when to progress the scenario by pressing a keyboard. The rest of the scenario then follows a similar structure of switching between interactive scenes and idle scenes with increasingly ‘difficulty’ as the stranger becomes increasingly confrontational during the interactive scenes (from commenting on the small size of the bench to directly confronting and asking the participant to leave). In the final confrontation, the participant is faced with a choice of telling the stranger and her friend to leave, or telling them that they can stay (figure 3). The purpose of this VE is to expose the patient to an informal situation with few expectations or social rules. Further, participants are exposed to social domains of informal interaction and assertiveness with progressive discomfort and finally direct confrontation, learning to endure and stand firm, as well as to teach the patient that even if an otherwise unlikely social conflict may occur and escalate, they can deal with it.

*New Employee:*In this scenario, the participant attends their first employee meeting at a new workplace. The scenario starts outside the conference room, in an empty hallway, where sounds of the colleagues’ voices can be heard through a partially open door. A textbox overlay then appears, prompting the participant to enter the room. Once oriented towards the textbox for 2 seconds, the scene cuts to a seated position around a breakfast table in a conference room with three coworkers. The coworkers greet the participant and then engage in small talk amongst each other (figure 4). Eventually the boss enters, and the meeting begins. The boss asks everyone to introduce themselves, ending with the participant’s introduction of him-/herself. After the coworkers’ presentations, the boss receives a phone call and leaves the room. As the boss enters and leaves the room, the social atmosphere changes from informal to formal and vice versa. Once the boss re-enters, the patient is tasked with presenting him-/herself by answering questions regarding workplace preferences, earlier experiences, hobbies, and private life. For each question posed, the clinician controls the reaction in body language from the coworkers and boss; positive, neutral, or negative (figure 5), by pressing one of three buttons on a keyboard after the participant is asked a question in VR. Once the participant is finished introducing themselves and answering questions, the boss leaves the room, while the coworkers go back to informal talk amongst themselves until the scenario ends a few minutes later. The purpose of this VE is to expose the patient to both informal and formal interaction, switching back and forth. Further, patients may be exposed to a variety of different expressions and body language (attentiveness and inattentiveness) or ambiguity by the surroundings in an ‘intimate’ presentation setting, with fewer people and questions ranging from professional to personal.

*Shopping:*

In this VE, the participant starts outside a grocery store. They are then presented with a textbox overlay, prompting the participant with the option of entering the store. Once oriented towards the textbox for 2 seconds, the scene cuts to just inside the entrance of the supermarket. Another textbox overlay then appears, prompting the participant to go collect the first item: flour. Once oriented towards the textbox for 2 seconds, the scene cuts to a vacant aisle with few people passing by – in front of the participant is flour. A textbox then appears, prompting the participant to pick up flour. Once fixated on for 2 seconds, the scene cuts to a similar scene in which flour is missing from the shelf and appeared in the shopping basket on the floor next to the participant. A textbox then appears (controlled by clinician), prompting the participant to move on and find the next item; toilet paper. Once fixated on for 2 seconds, the scene cuts to a slightly busier aisle. Ahead of the participant is an employee stocking toilet paper on a pallet. The participant is then presented with two overlay textbox options; 1) “Contact the employee” or 2) “Wait for the employee to finish and grab one”. If the contact option is chosen, the participant will then do so verbally and the practitioner will cut to the scene in which the employee has been contacted (figure 6). If the waiting option was chosen, the clinician cuts to a scene in which the employee finishes stacking up the toilet paper and leaves. The participant is then prompted with a textbox to go find the last item; eggs. Once selected, the scene cuts to the participant standing in the refrigerated aisle, with eggs ahead of them. A textbox then appears, prompting the participant to collect the eggs. Once chosen, the scene cuts and the eggs slide off the shelf and onto the floor in front of the participant. Bystanders react to this and the participant is then prompted with a choice of two textboxes indicating: 1) contact an employee; 2) go to checkout. This scenario is created to induce progressive discomfort for each item. Levels of difficulty increases between each picked up item. The practitioner can chose to give the textbox option of going to checkout after each item. The difficulty of the situation at checkout reflects the difficulty of the most recent situation (e.g., if the participant only picked up flour, the experience at the register is smooth with no other costumers in line; if the participant went all the way through and picked up all three items, the experience is less smooth and problems with grocery scanner, cash register, and complaining from people in line occurs (figure 7)). The purpose of this VE is to expose the patient to being observed by others, initiating contact, being at the center of attention in a public setting, having an accident in a public setting.

*Presentation:*
The VE starts outside the conference room, in an empty hallway, where sounds of voices can be heard through a partially open door. The participant enters the room by orienting towards a textbox prompt for two seconds. The scene then cuts to a medium-large conference room with desks in a U-formation. After initial small talk amongst the crowed, everyone gets seated. The participant is seated in the middle (bottom of the U). The crowd continues to small talk until the chairperson arrives (figure 8). The chairperson welcomes everyone and announces; “*We’ll start out the day with a presentation, and today, the presentation is given by you*”. Attendees then look towards the participant, as the chairperson asks the participant if they would like to give the presentation seated at the table, or standing by the blackboard. The chairperson does not mention a subject or theme, nor is the context of the meeting clarified. In this experiment, the subject of presentation was chosen by the participant beforehand, but not practiced. Depending on the participant’s choice of standing (figure 9) or sitting, the crowd is either neutral or positive in their expressions, body language, questions after the presentation, and comments during the presentation (e.g., asking the participant to speak louder). During the presentation, the scenario loops after 8 minutes. Once the participant has finished their presentation, the clinician decides when to proceed to the next scene. The following scene includes a question from the audience pertaining to the rationale for choosing their subject, followed by another idle scenario. The clinician then decides when to proceed to the final scene in which the participant is given thanks as the chairperson moves the meeting along. The scenario then ends. The purpose of this VE is to practice being observed by others in a formal setting, whilst being at the center of attention. Further, as the VE was designed to be applicable to a presentation on any subject, patients can practice presentation performance with little to no preparation if wished.

## Figures


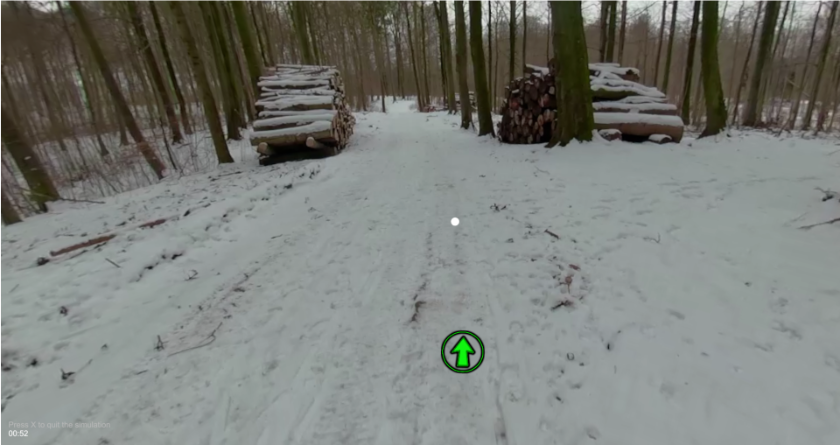


Figure 3: The baseline scenario


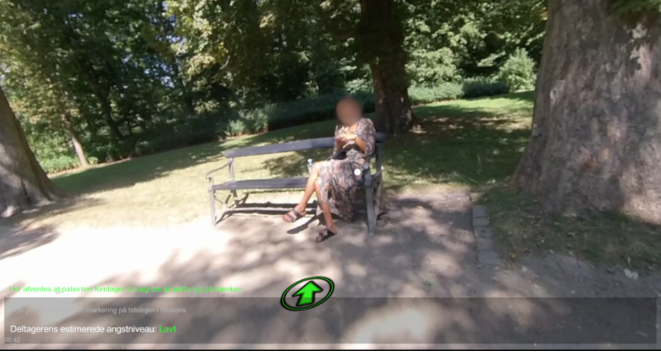


Figure 4: Bench scenario. Starting position


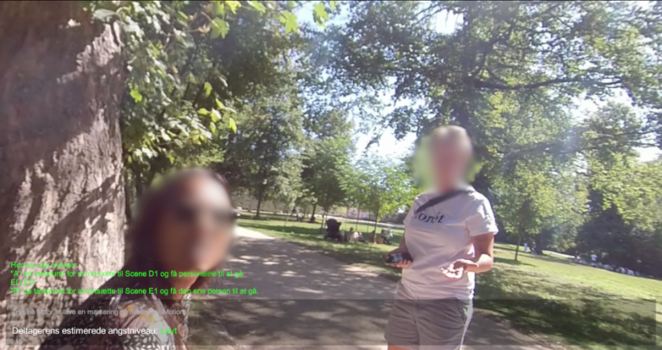


Figure 5: Bench scenario. Participant is given the choice of asking the strangers to leave or stay.


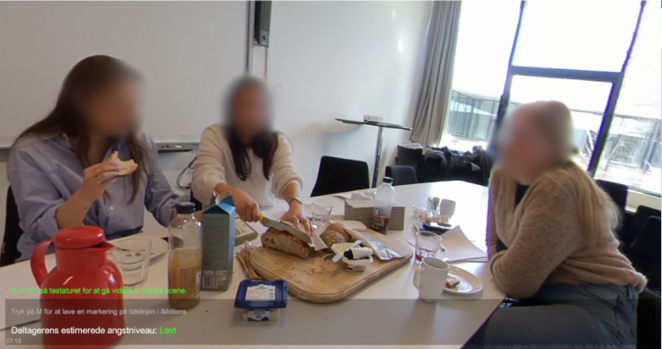


Figure 6: New Employee scenario. Informal conversation around the table.


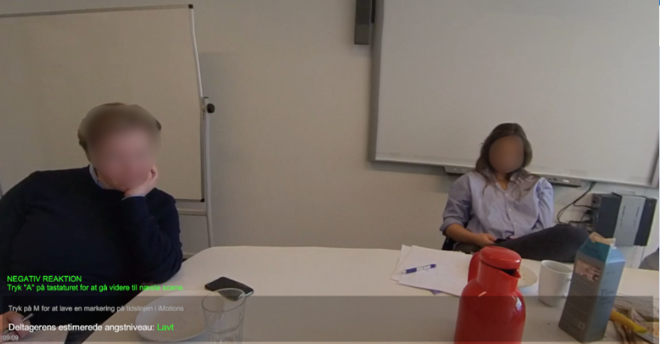


Figure 7: New Employee scenario. Replying to the boss’ questions. Negative reaction


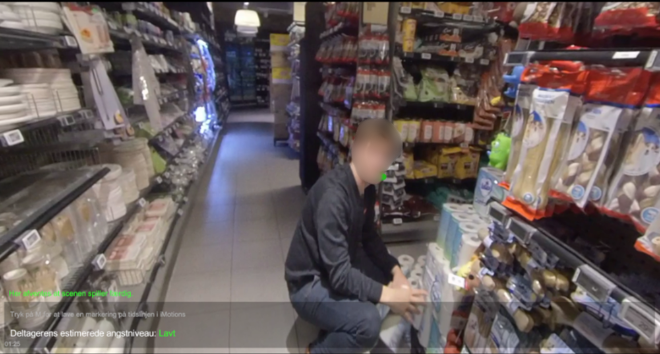


Figure 8: Shopping scenario. Contacting an employee


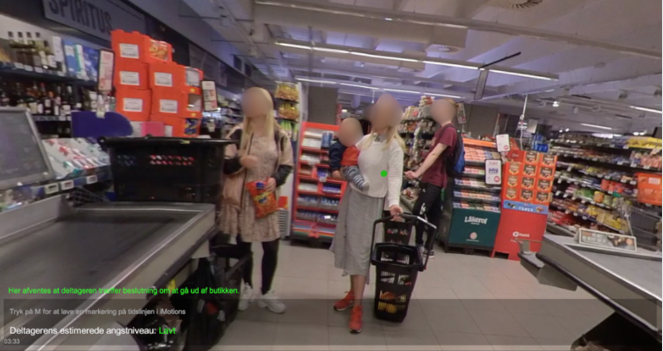


Figure 9: Shopping scenario. Checkout at the cash register.


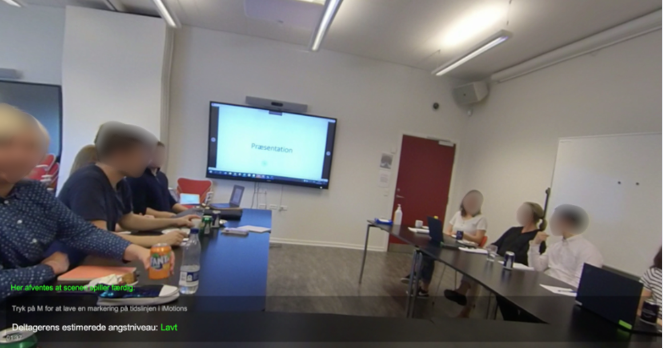


Figure 10: Presentation scenario. Waiting for the chairperson to arrive.


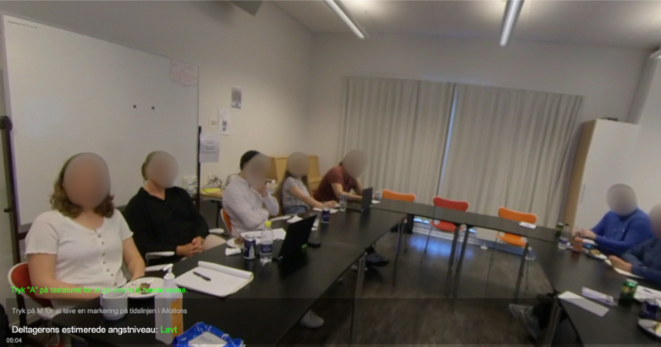


Figure 11: Presentation scenario. Presenting a subject, standing.
